# *Se14*, Encoding a JmjC Domain-Containing Protein, Plays Key Roles in Long-Day Suppression of Rice Flowering through the Demethylation of H3K4me3 of *RFT1*

**DOI:** 10.1371/journal.pone.0096064

**Published:** 2014-04-23

**Authors:** Takayuki Yokoo, Hiroki Saito, Yoshihiro Yoshitake, Quan Xu, Takehito Asami, Takuji Tsukiyama, Masayoshi Teraishi, Yutaka Okumoto, Takatoshi Tanisaka

**Affiliations:** Graduate School of Agriculture, Kyoto University, Kyoto, Japan; Chiba University, Japan

## Abstract

Floral transition from the vegetative to the reproductive growth phase is a major change in the plant life cycle and a key factor in reproductive success. In rice (*Oryza sativa* L.), a facultative short-day plant, numerous flowering time and flower formation genes that control floral transition have been identified and their physiological effects and biochemical functions have been clarified. In the present study, we used a *Se14*-deficient mutant line (HS112) and other flowering mutant lines to investigate the photoperiodic response, chromosomal location and function in the photoperiod sensitivity of the *Se14* gene. We also studied the interactive effects of this locus with other crucial flowering time genes. We found that *Se14* is independent of the known photoperiod-sensitive genes, such as *Hd1* and *Ghd7*, and is identical to Os03g0151300, which encodes a Jumonji C (JmjC) domain-containing protein. Expression analysis revealed that the expressions of *RFT1*, a floral initiator known as a “florigen-like gene”, and *Ehd1* were up-regulated in HS112, whereas this up-regulation was not observed in the original variety of ‘Gimbozu’. ChIP assays of the methylation states of histone H3 at lysine 4 (H3K4) revealed that the trimethylated H3K4 in the promoter region of the *RFT1* chromatin was significantly increased in HS112. We conclude that *Se14* is a novel photoperiod-sensitivity gene that has a suppressive effect on floral transition (flowering time) under long day-length conditions through the modification of chromatin structure by H3K4me3 demethylation in the promoter region of *RFT1*.

## Introduction

Floral transition from the vegetative to the reproductive growth phase is a major change in the plant lifecycle that is critical to reproductive success. In rice (*Oryza sativa* L.), a facultative short-day plant, floral transition is promoted under short day-length conditions and delayed under long day-length conditions. To date, numerous flowering time and flower formation genes that control the floral transition of rice have been identified [1]–[4], and the physiological effects and biochemical functions have already been identified for many of these genes. Based on their physiological effects and interactions with other genes, several genetic pathways to floral transition have been proposed [5]–[7].

*Early heading date 1* (*Ehd1*) functions as a flowering time activator under both short and long day-length conditions [2], and regulates the duration of the basic vegetative growth period [8]. *Ehd1* promotes the expression of two florigen-like genes, *Heading date 3a* (*Hd3a*) and *Rice FT-like 1* (*RFT1*), and thereby accelerates flowering. Under long day-length conditions, the expression of *Ehd1* is suppressed by *Grain number, plant height and heading date 7* (*Ghd7*), which encodes a CCT (CO, CO-like, and TIMING OF CAB1)-domain protein [4]. *Ghd7* acts as a key factor in the photoperiodic pathway to flowering and delays flowering under long day-length conditions through inhibiting *Ehd1* expression. Under long day-length conditions, *Ghd7* is up-regulated by gated signaling of red light [9]. We previously reported that *Photoperiod sensitivity-13* (*Se13*) encodes phytochromobilin synthase involved in phytochrome activity [10]. The *Se13*-deficient mutants flowered extremely early (about 40 days earlier than the original variety) even under long day-length conditions due to the lack of functional phytochromes (Yoshitake et al., unpublished). Such a red-light-dependent suppression pathway(s), including *Ehd1* and *Ghd7*, does not exist in *Arabidopsis* and is considered specific to rice. In addition to *Ghd7*, *OsMADS50* and *Ehd2* are known to be activators of the florigen-like genes *Hd3a* and *RFT1* through the up-regulation of *Ehd1* expression, thereby promoting flowering under long day-length conditions [3], [11]. On the other hand, *Heading date 1* (*Hd1*) is another key regulator that controls *Hd3a* expression in the photoperiodic pathway, independent of *Ehd1*, and delays and slightly promotes flowering under long and short day-length conditions, respectively [12], [13].

Chromatin structure allows accessibility of factors and cofactors that regulate gene expression for biological processes of plant development including floral transition. Histone lysine methylation is an essential epigenetic modification of chromatin structure having activating and suppressing effects on gene expressions [14]. The methylation of histone H3 lysine 4 (H3K4) and lysine 36 (H3K36) primarily has an activating effect on gene expression, whereas that of histone H3 lysine 9 (H3K9), lysine 27 (H3K27), and histone H4 lysine 20 (H4K20) has a suppressing effect on gene expression [15].

It has been suggested that Jumonji C (JmjC) domain-containing proteins function as histone demethylases [16]. These proteins are capable of demethylating all of the mono-, di- and tri-memethylated lysines of histones [17]. Recent studies on *Arabidopsis thaliana*, a facultative long-day plant, have shown that the expression of *FLOWERING LOCUS C* (*FLC*) and *FLOWERING LOCUS T* (*FT*) are regulated through chromatin modifications. *FLC* is epigenetically regulated by *ARABIDOPSIS TRITHORAX 1* (*ATX1*) and the *EARLY FLOWERING IN SHORT DAYS* (*EFS*) which mediate H3K4 and H3K36 methylation, respectively, and act as inhibitors of *Arabidopsis* flowering [18]–[20]. *Arabidopsis thaliana Jumonji 4* (*Atjmj4*) and *EARLY FLOWERING6* (*ELF6*), both of which encode JmjC-domain containing proteins, function as H3K4 demethylases of *FT* histones and repress *FT* expression [21].

In rice, JmjC domain-containing proteins are also conserved and some of these have been identified. JMJ706, a rice member of the JMJD2 family of JmjC genes [17], is involved in H3K9 demethylation, which is required for the expression of a subset of regulatory genes for rice floral development [22]. JMJ703 is a histone H3K4-specific demethylase and is responsible for a unique mechanism of controlling retrotransposon activity, which further strengthens the link between epigenetic silencing and genome stability [23].

Our previous study indicated that the early flowering of a mutant line HS112 was conferred by a single recessive mutant gene, *photoperiod sensitivity-14* (*se14*) [24]. In the present study, we investigated the photoperiodic response, chromosomal location and function of the *Se14* locus, including the interactive effects of this locus with other crucial flowering time genes. We found that the functional allele *Se14* at the *Se14* locus is a unique photoperiod-sensitivity gene that encodes a JmjC domain-containing protein with a zinc-finger (ZnF) domain, and functions as a demethylase of H3K4 methylation of the *RFT1* chromatin, resulting in delayed flowering under long day-length conditions.

## Results

### Analysis of Photoperiodic Response

**Figure 1:**
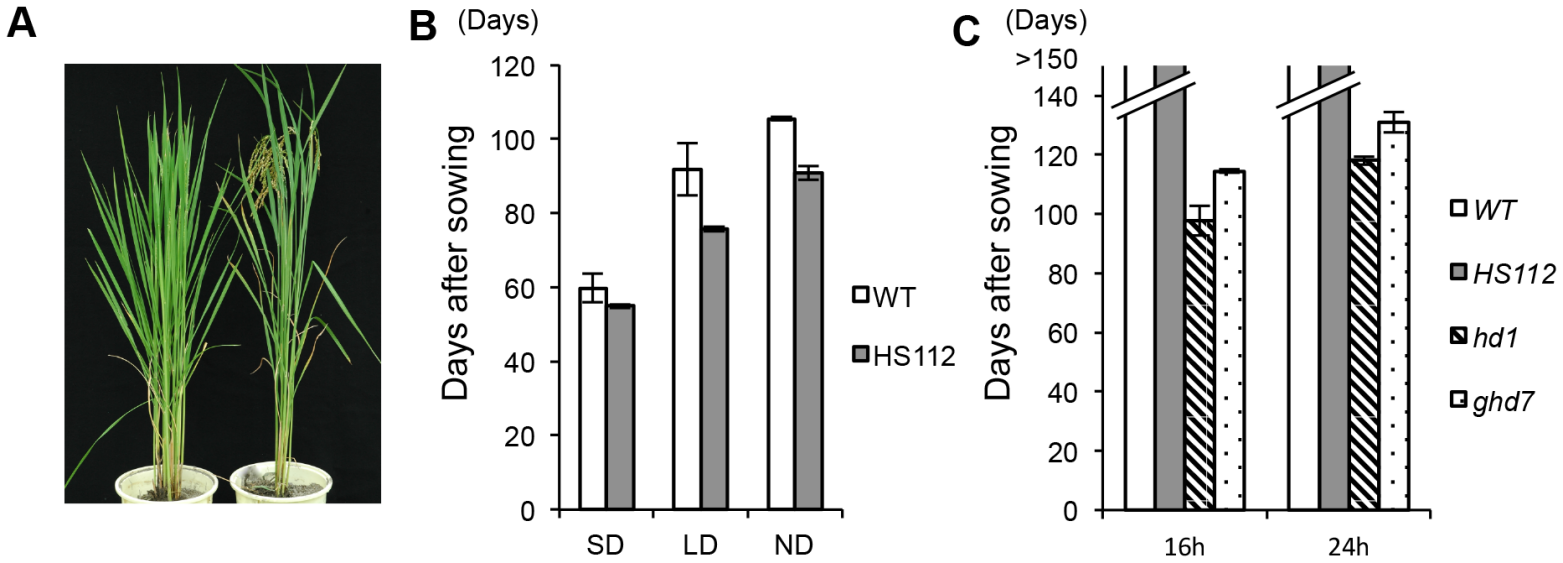
Phenotype of early flowering mutant HS112. A) 100-day-old plants of the wild type Gimbozu (WT; left) and HS112 (right) grown under normal day-length conditions (ND) in Kyoto. B) Days to heading (DH;  =  flowering time) of HS112 and the WT under 10 h (SD: short day-length), 14.5 h (14.5 LD: 14.5 h long day-length) and ND conditions. C) DH of HS112, *hd1* and *ghd7* under 16 h (16 LD: 16 h long day-length) and 24 h (24 LD: continuous light) conditions. Error bars indicate standard deviations.

HS112 is an early flowering time mutant, which was induced by gamma-ray irradiation of seeds of the *japonica* rice variety Gimbozu (WT; Figure 1A). The early flowering of this mutant line has proved to be conferred by a single recessive mutant allele *se14* at the *Se14* locus [24]. We examined the days to heading (DH;  =  flowering time) of HS112 and the WT under the following five day-length conditions: 10 h (SD), 14.5 h (14.5 LD), 16 h (16 LD), 24 h (24 LD), and a natural day-length (ND) in our experimental field in Kyoto, Japan (NL35°01′). In addition, we examined the DH of two single mutant lines, *hd1* and *ghd7*, which harbor recessive mutant alleles at the *Hd1* and *Ghd7* loci, respectively, under 16 LD and 24 LD conditions. Under SD, HS112 (54.9 DH) showed almost the same DH as that of WT (59.8 DH). Under 14.5 LD, however, HS112 exhibited 75.7 DH, while WT (91.8 DH) flowered two weeks later than HS112 (Figure 1B). Under ND, both HS112 and WT exhibited similar DH to plants under 14.5 LD, respectively. In Kyoto, the natural day-length is longer than the critical day-length (approximately 13.5 h) and does not exceed 14.5 h during the cropping season from May to the end of July [25]. This is regarded as a long day-length condition. After July, it becomes shorter than 13.5 h, which is regarded as a short day-length condition [10]. Thus, the ND and 14.5 LD used in this study were considered to be almost the same photoperiod. Under 16 LD and 24 LD conditions, neither the WT nor HS112 flowered even after 150 days from sowing (Figure 1C). In contrast, the *hd1* and *ghd7* mutants flowered 115 and 130 days after sowing, respectively. These results indicate that the *Se14* locus is involved in the photoperiodic pathway to flowering, but the role of the *Se14* locus is different from the roles of the *Hd1* and *Ghd7* loci.

### Identification of Chromosomal Location and the Candidate Gene of *Se14*

**Figure 2:**
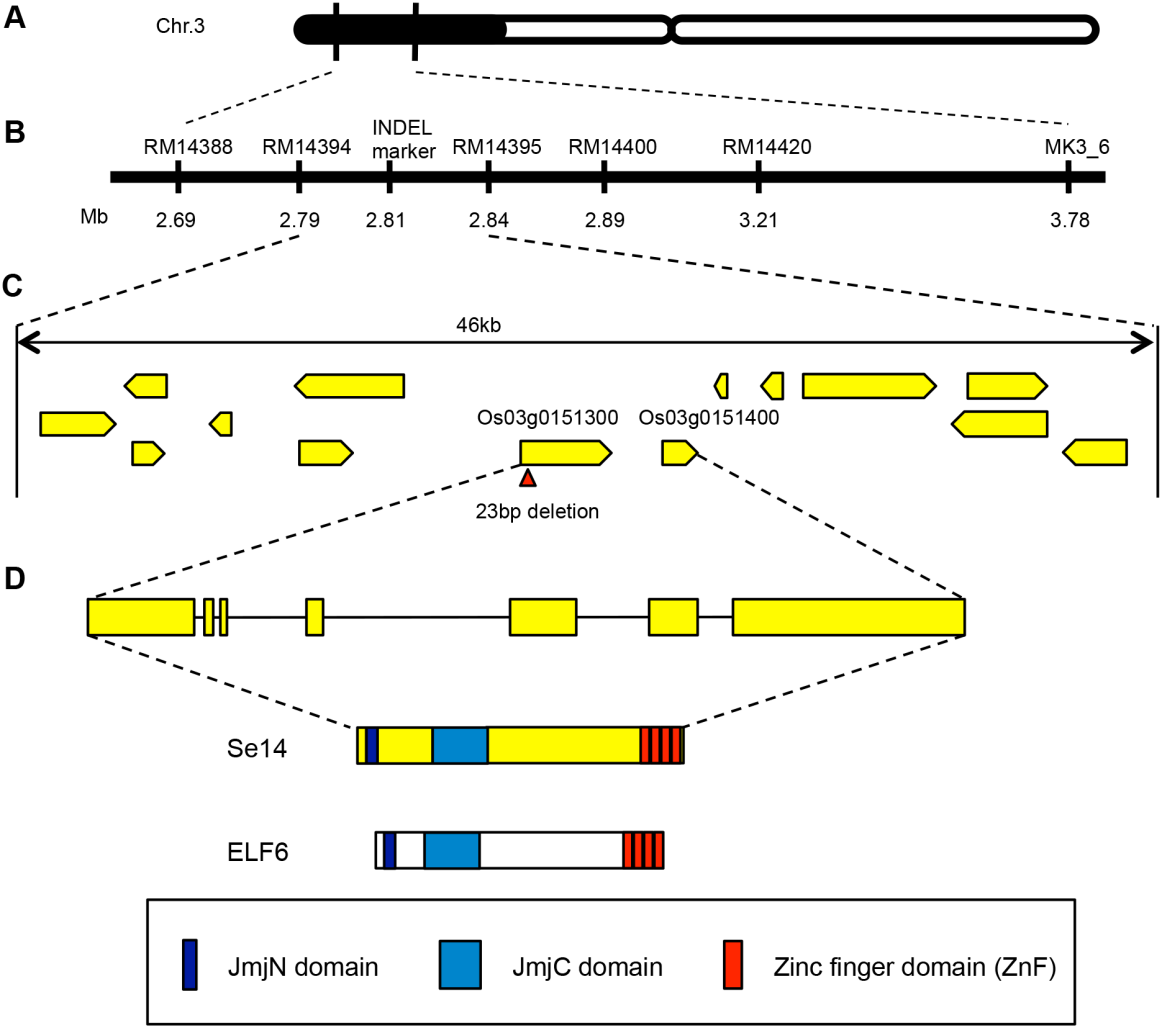
Map-based cloning of *Se14* and structural comparison of the proteins between Se14 and ELF6. A) Chromosomal location of *Se14* and marker positions on chromosome 3. B) High-resolution linkage map of *Se14* and genes annotated in the Rice Annotation Project Database (RAP-DB; http://rapdb.lab.nig.ac.jp). C) Genomic structure of the candidate region and the mutation in HS112. D) Schematic domain structure of two loci, Os03g0151300 and Os03g0151400, annotated at RAP-DB, and their combined cDNA.

We attempted to perform a fine mapping of the *Se14* locus using the F_2_ population, comprisied of 96 plants, from a cross between HS112 and a chromosome segment substitution line SL13 harboring a Kasalath-derived chromosome segment including the candidate region of the *Se14* locus on the short arm of chromosome 3, on a Nipponbare background [26]. Experimental results demonstrated that the *Se14* locus was located in a region with a physical distance of approximately 1 Mb between the two markers, RM14388 and MK3_6, on chromosome 3. Further analysis was conducted using 3920 F_3_ plants from the recombinants (F_2_ plants) of RM14388 and MK3_6, with five additional SSR markers and one INDEL marker. Consequently, the *Se14* locus was narrowed down to the region between the two SSR markers, RM14394 and RM14395, with a physical distance of less than 46 kb (Figure 2B). According to the Rice Annotation Project Database (RAP-DB) (http://rapdb.lab.nig.ac.jp), this 46 kb genomic region contains 14 genes supported by full-length cDNA. We analyzed the nucleotide sequences of these regions of WT and HS112, and found no mutations in HS112 except for a 23 bp deletion at Os03g0151300, which encodes a Jumonji C (JmjC) domain-containing protein. This deletion was located in the first exon, resulting in a frame-shift mutation spoiling all the functional domains by producing a premature stop codon (Figure 2C). It is therefore considered that Os03g0151300 is a likely candidate for the *Se14* gene. To determine the cDNA sequence, we performed 5′-RACE and 3′-RACE experiments using Os03g0151300-specific primers. Interestingly, the experimental results showed that in addition to the cDNA sequence of Os03g0151300, the transcript included four additional exons whose sequences were partially identical with those of the cDNA of Os03g0151400, located next to Os03g0151300 (Figure 2D). The combined cDNA encodes a protein containing two functional domains, Jumonji N (JmjN) and JmjC, with four copies of C2H2-type zinc finger (ZnF) domains, which share high homology with *Arabidopsis* ELF6 (Figure 2D and Figure S1). This indicates that *Se14* is most likely Os03g0151300, and the spoiling of functional domains due to the frame-shift mutation of Os03g0151300 causes the decrease of the photoperiod sensitivity of HS112.

### Analysis of Interactions of the *Se14* Locus with the *Ehd1*, *Se13*, *Hd1* and *Ghd7* Loci

**Figure 3:**
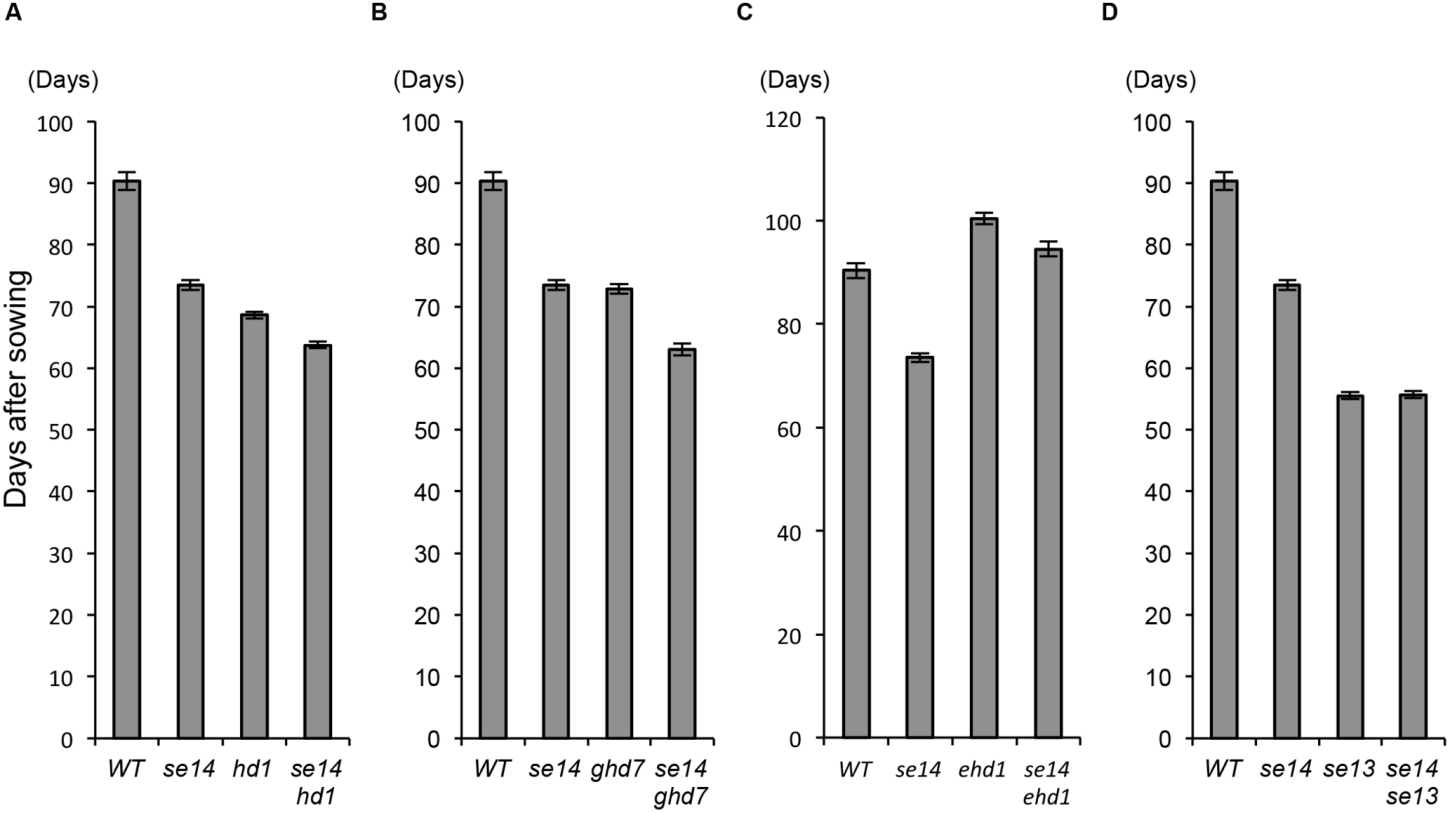
Genetic interaction of the *Se14* locus with other flowering time genes loci. Comparison of flowering time among single mutant lines for flowering time: A) *hd1*, B) *ghd7*, C) ehd1 and D) *se13*, and their double mutant lines for *se14*. These lines were grown under ND.

Interactions of the *Se14* locus with other flowering time loci were investigated using four single mutant lines, *ehd1*, *hd1*, *ghd7* and *se13*, and four double mutant lines, *se14 ehd1*, *se14 hd1*, *se14 ghd7* and *se14 se13*. These lines were grown under ND conditions in Kyoto. The double mutant lines, *se14 hd1* and *se14 ghd7*, flowered earlier than their respective single mutant lines (Figure 3A and 3B). In addition, the double mutant lines, *se14 ehd1*, flowered intermediately between their respective single mutant lines (Figure 3C). This indicates that the functional allele *Se14* at the *Se14* locus suppresses flowering independently of *Ehd1*, *Hd1* and *Ghd7*. On the other hand, there was no significant difference in DH between the double mutant line *se13 se14* and its single mutant line *se13*, suggesting that the functional allele *Se14* does not affect flowering time in an *Se13*-deficient genetic background (Figure 3D). *Se13* encodes phytochromobilin synthase, which is involved in phytochrome activity [10], and the *se13* mutant flowered extremely early even under long day-length conditions. Therefore, *Se14* might be involved in the suppression pathway regulated by the red-light signal.

### Expression Analyses of Flowering Time Genes in HS112

**Figure 4:**
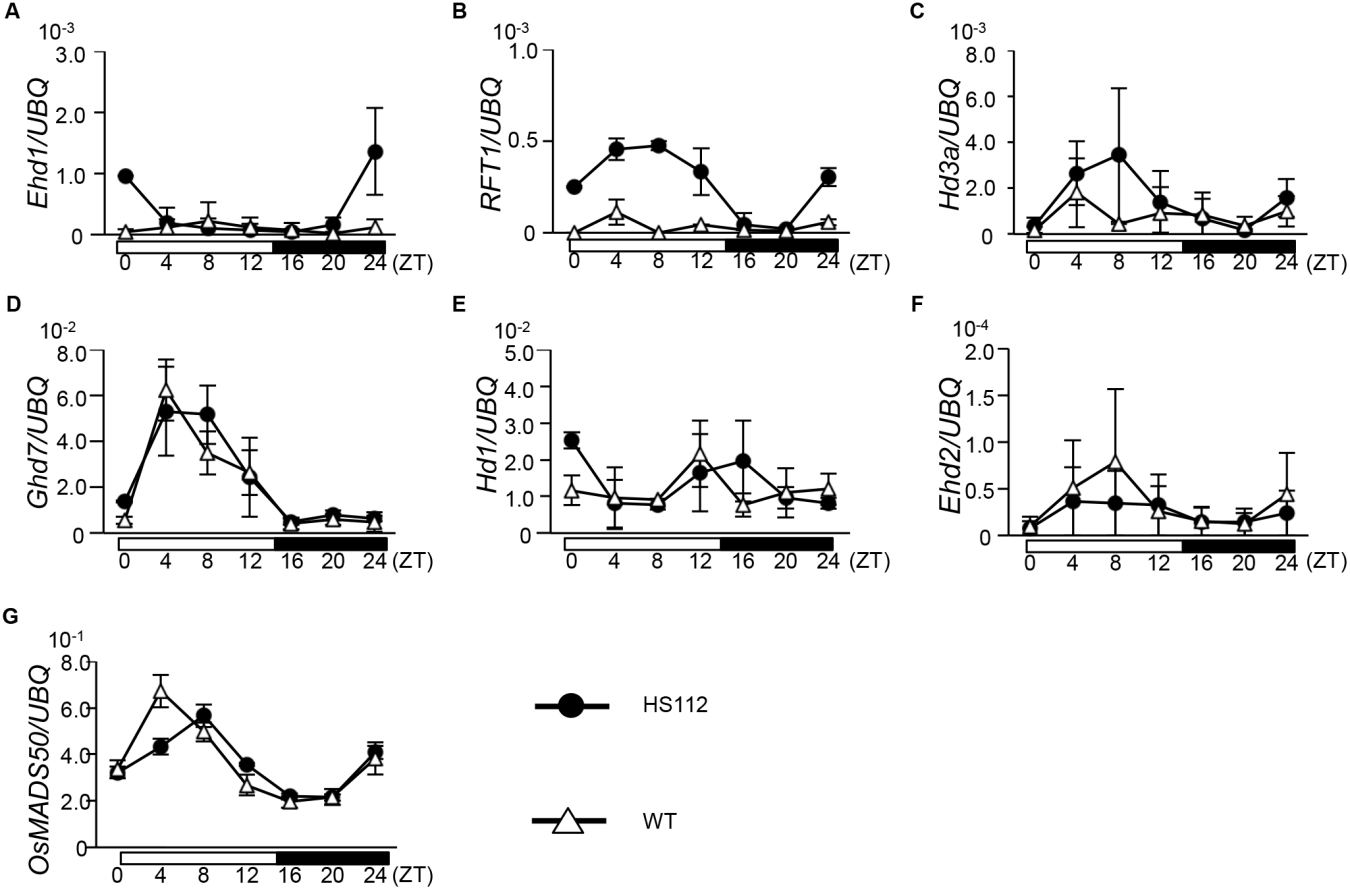
Diurnal expression of flowering time genes in the WT and HS112. Transcriptional level of the major flowering time genes were compared between HS112 (*se14*) and Gimbozu (WT) under 14.5 h day-length conditions. X axis means Zeitgeber time (ZT), and the black bars indicate the dark period, and the white bars indicate the light period. Thirty days after sowing, leaves of three plants were sampled at 4 h intervals (three replications). Expression analysis was performed by the standard curve method. For comparing expression levels among the genes, the relative expression level of each gene against the UBQ expression level was calculated.

The diurnal expression of flowering time genes under a 14.5 h day-length condition (a long day-length condition) was analyzed to elucidate the molecular regulation of early flowering of HS112 conferred by *se14*. The expression of *Ehd1* was increased in HS112 at night, but this increase was not observed in the WT (Figure 4A). The expression of *RFT1* was increased in HS112, except during early night, while the WT showed lower expression almost throughout the day (Figure 4B). The expression of *Hd3a* was somewhat elevated during daytime in HS112, but there was no significant difference between HS112 and the WT (Figure 4C). On the other hand, no significant difference was observed in the expression of *Ghd7* and *Hd1* between HS112 and the WT (Figure 4D and 4E). Interestingly, there was no apparent difference in the expression of *OsMADS50* and *Ehd2*, both of which are positive regulators of *Ehd1*, between HS112 and the WT (Figure 4F and 4G). These results indicate that the *Se14* directly or indirectly suppresses the expression of *RFT1* and *Ehd1*.

### Investigation of H3K4 Methylation States in HS112

**Figure 5:**
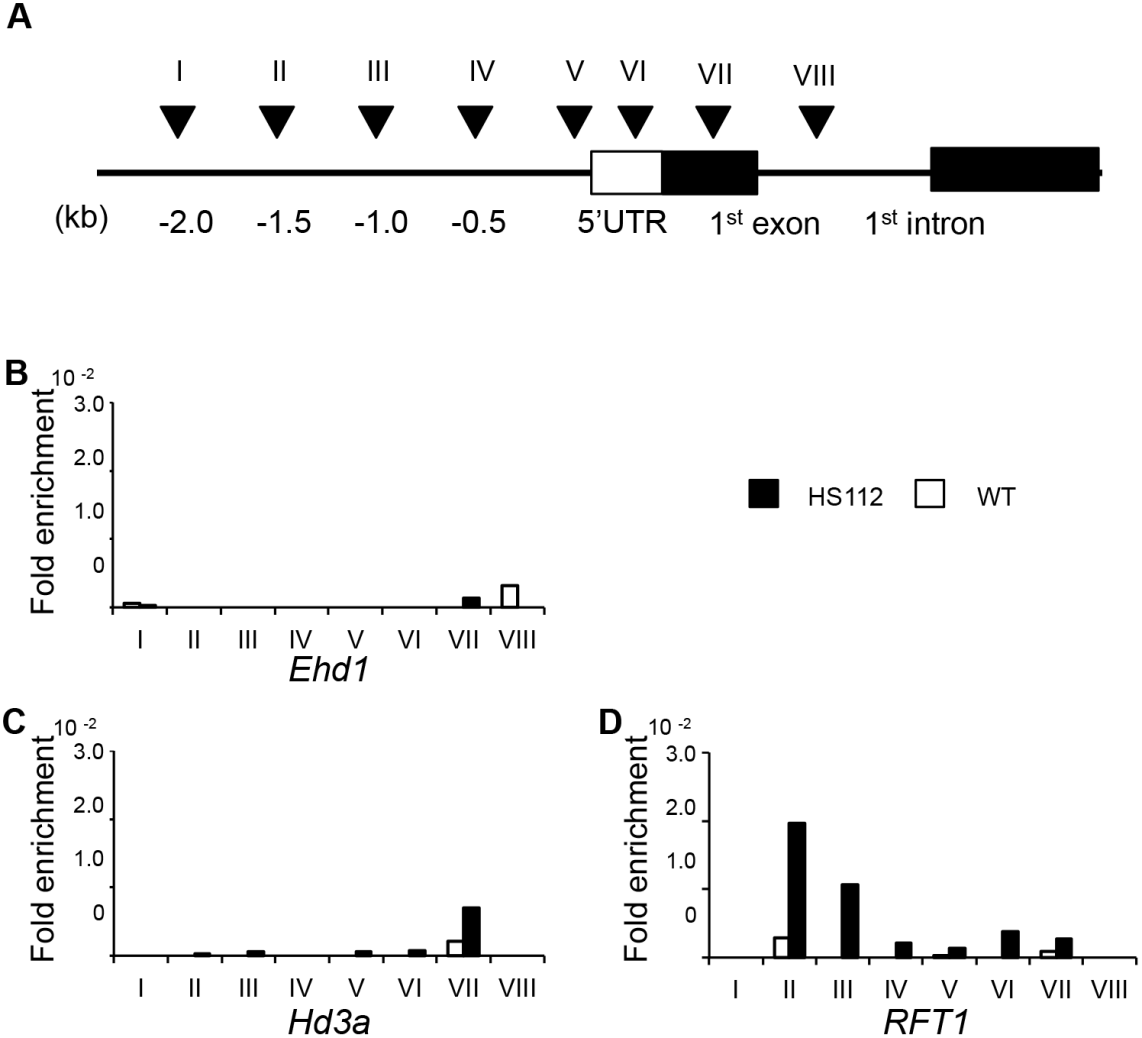
Histone methylation levels at *Ehd1*, *Hd3a* and *RFT1.* A) The location of primer sets used for ChIP assay. A total of five primer sets named I to V were prepared on the promoter region every 500 bp from −2.0 kb to 0 kb at in the upstream region of TSS, and three sets named VI to VIII on 5′ UTR, exon 1 and intron 1, respectively. B∼J) Relative levels of H3K4me3 in *Ehd1, Hd3a* and *RFT1* chromatin. The amount of DNA fragments of the ChIP assay were quantified with three replications of real-time PCR. Concentrations of each sample were normalized to those manipulated with H3 universal antibody.

Histone methylation marks are generally associated with transcriptional chromatin states. According to the predicted amino acid sequence, the Se14 protein is expected to function as an H3K4 demethylase. To confirm this, we investigated the deposition of histone methylation marks on the chromatins of *Ehd1*, *Hd3a* and *RFT1* by chromatin immunoprecipitation (ChIP) assays covering every 500 bp region 2 kb-upstream of the transcription start site (TSS) and the coding region (Figure 5A). Plants of HS112 were grown under the same experimental conditions as those in expression analysis, 14.5 h day-length (14.5 h light, 30°C/9.5 h dark, 25°C) at 70% relative humidity. Thirty days after sowing, we collected fully opened leaves from the top of seedlings. Experimental results showed that the H3K4me3 levels were significantly increased in the II and III regions of the *RFT1* chromatin in HS112, while those were slightly increased in the coding region (Figure 5D). In coding region of *RFT1*, the H3K4me3 levels were slightly increased (Figure 5D). On the other hands, the H3K4me3 level in the chromatin regions including the both of promoter and coding regions of *Ehd1* and *Hd3a* did not significantly differ between HS112 and the WT (Figures 5B and C). These results suggest that Se14 functions as a demethylase of the H3K4 tri-methylation mark in the *RFT1* chromatin region.

## Discussion

In this study, we confirmed that the early flowering of the mutant line HS112 is caused by a single recessive mutant gene *se14* at the photoperiod sensitive locus *Se14*. Analysis of photoperiodic response showed that the *Se14* locus is involved in the photoperiodic pathway in rice, but the role of this locus differs from the roles of two other crucial loci in the photoperiodic pathway, *Hd1* and *Ghd7*. Subsequent linkage analysis revealed that the *Se14* locus is between RM14394 and RM14395, in a region with a physical distance of less than 46 kb on chromosome 3 (Figure 2B). A database search for this region showed that Os03g0151300 is a candidate for the *Se14* gene. As a result of sequence analysis of the candidate region, HS112 was found to harbor a 23 bp deletion in exon 1 of Os03g0151300, which causes a frame-shift mutation producing a premature stop codon in all of the functional domains (Figure 2C). Therefore, we conclude that the *Se14* locus is identical to the Os03g0151300 locus, and *se14* is a loss-of-function allele at the *Se14* locus. The *Se14* gene encodes a JmjN and JmjC domain-containing protein with four copies of the C2H2-type ZnF domain. ChIP assays showed that trimethylated H3K4 of HS112 was specifically increased at the upstream region of the *RFT1* chromatin (Figure 5D). Thus, *Se14* is considered to be a key gene in controlling H3K4 trimethylation states in the *RFT1* chromatin, thereby acting as a suppressor of *RFT1* under long day-length conditions.

Chromatin structure is important for the accessibility of factors and cofactors that regulate gene expressions. Histone lysine methylation has garnered a lot of attention due to the complicated role it plays in the epigenetic modification that both activates and suppresses gene expression [14]. The methylation state of H3K4 primarily has an activating function of gene expression involved in flowering time control [15], [27]. Recent studies on *Arabidopsis* have suggested that JmjC domain-containing proteins function as histone demethylases [16]. They are capable of demethylating all of the mono-, di- and tri-memethylated lysines of histones [17]. It has been reported that *Atjmj4* and *ELF6*, which encode a JmjC-containing protein resembling those of the human JARID1 family, function as H3K4 demethylases of *FT* histones and suppress *FT* expression [21]. Yang et al. (2012) reported that overexpression of JMJ15, which is a member of the H3K4me3 demethylase JARID1 family, resulted in obvious early flowering due to the reduction in H3K4me3 at the *FLC* locus associated with the suppression of *FLC* transcription levels [28]. It is therefore believed that JmjC domain-containing proteins are crucial factors for mediating the H3K4me state and regulating gene expression involved in flowering time control.

Chen et al. (2013) demonstrated that, in rice, a loss-of-function mutation of a JmjC domain-containing protein, JMJ703, affected stem elongation and plant growth, which may be related to the increased expression of cytokinin oxidase genes due to failure of demethylation of H3K4me marks [29]. Therefore, the H3K4me mark is important for the epigenetic remodeling of the chromatin structure that regulates rice growth. Genome-wide analyses of H3K4 methylation states in Arabidopsis and rice showed that H3K4 methylation marks are found to be almost exclusively genic, and that H3K4me2 and H3K4me3 accumulate predominantly in promoters and 5′ genic region [30], [31]. On the other hand, Li et al (2008) reported that 25.3% of H3K4me2 and 27.2% of H3K4me3 were found in the region without a gene body (annotated transcribed region) plus its putative promoter (the 1 kb region upstream of the annotated transcription start site [TSS]) [32]. In human, it is reported that H3K4 states at the 1 kb upstream region from TSS is strongly associated with gene expression [33]. Therefore, we concluded that the high H3K4me3 level over 1 kb upstream region (position II and III in Figure 5) from TSS of *RFT1* is essential to up-regulate the expression. Our study is the first to demonstrate that JmjC domain-containing proteins regulate the H3K4me state associated with the expression of *RFT1*. Thus, the epigenetic modification of JmjC domain-containing proteins based on the H3K4me state is a key regulatory system for flowering time control in rice.

It is noteworthy that the H3K4me3 states of *Ehd1* and *Hd3a* in HS112 were not different from those of the WT (Figure 5). This indicates that the *Se14* demethylation is specific to the H3K4me state of *RFT1*. *Hd3a* and *RFT1* are paralogs and members of the phosphatidylethanolamine-binding protein gene family [34]. The function of the two paralogs, however, appears to have diverged after duplication [35]. It has been shown that *Hd3a* and *RFT1* are the major activators of flowering under short and long day-length conditions, respectively [36]–[38]. Furthermore, the nucleotide sequences of *RFT1*, compared with those of *Hd3a*, are highly diversified among varieties, suggesting that *RFT1* has diverged more rapidly than *Hd3a* during rice evolution [35]. Taken together, it appears that the selectivity based on histone modification by *Se14* might have different regulatory actions dependent upon photoperiod, resulting in the long-day suppression of flowering.

**Figure 6:**
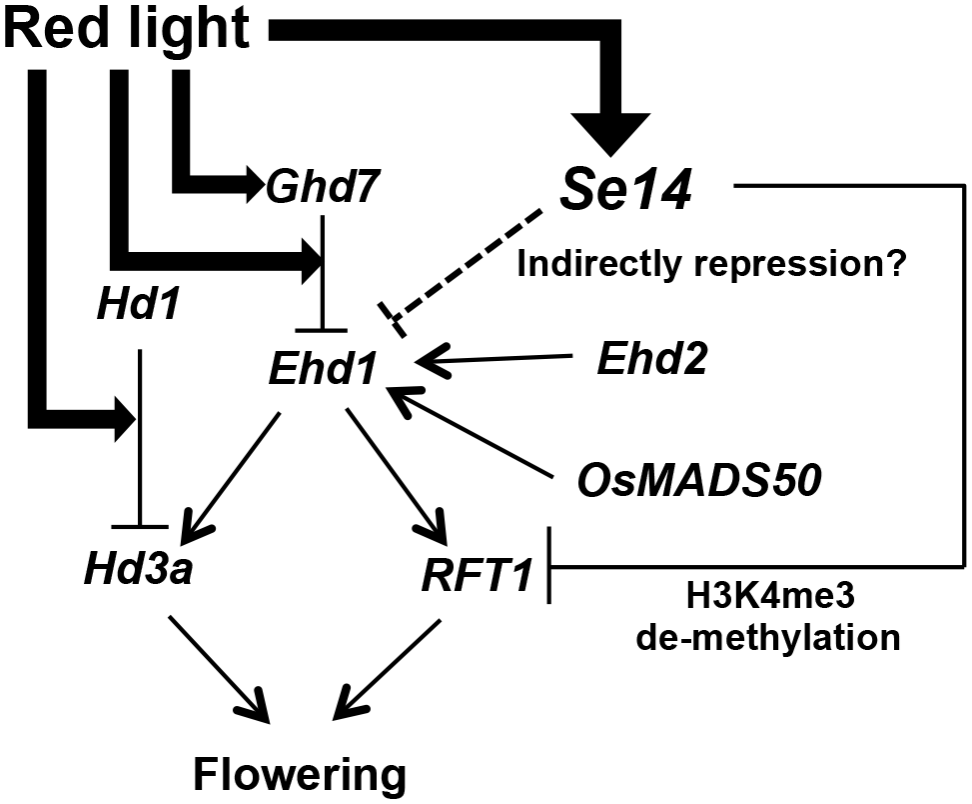
Model of long-day suppression of flowering time in rice. Solid line indicates direct regulation, and dotted line indicates indirect regulation. *Se14*, which is up-regulated by red-light, delays flowering time due to down-regulation of *RFT1* expression by demethylation of the H3K4me state. *Se14* also suppresses the expressions of *Ehd1,* which is independent with the regulation of Ghd7 protein mediated by red-light, resulting in delayed flowering time.

Analyses of interallelic interactions and gene expression indicated that *Se14* regulates flowering time independently of *Hd1* and *Ghd7* (Figures 3 and 4). Itoh et al. (2010) demonstrated that *Ghd7* is regulated by gated phytochrome signaling and repressed the expression of *Ehd1* [9]. In present study, the expression of *Ehd1* was up-regulated in HS112, in spite of high expression of *Ghd7*. In addition, the H3K4 tri-methylation states of *Ehd1* were not changed in HS112. Therefore, *Se14* might be indirectly involved in the repression of *Ehd1* independent with the pathways of *Ghd7* (Figure 6). Further, the analysis of interallelic interaction between *Se14* and *Ehd1* suggested that *Se14* represses flowering independently of *Ehd1*. Thus, it is indicated that *Se14* is more effective to delay flowering through the repression of *RFT1* by H3K4me3 demethylation than the indirect repression of *Ehd1*. On the other hands, the analysis of interallelic interaction between *Se14* and *Se13* demonstrated that *Se14* did not function in the *Se13*-deficient genetic background (Figure 3C). Interestingly, the expression of *Se14* was down-regulated in the *Se13*-deficient genetic background (Figure S2). Jang et al. (2011) demonstrated that the repression of *phyA* by light is correlated with alternations in specific histone marks [39]. In the dark, methylation of H3K4me3 were detected at the active *phyA* locus, whereas upon light treatment, increased H3K27me3 mark and decreased H3K4me3 mark were associated with the repressed gene [39]. They also reported that the chromatin modifications were blocked in the *phyB-9* mutant in red light, indicating that these changes are mediated by the *phyB* signaling pathway [39]. Our study also showed that the diurnal expression of *Se14* was highly transcribed during the light period (Figure S2). It is therefore suggested that the *Se14* might be mediated by the red-light signal (Figure 6). Further analysis is necessary to clarify the light-mediated H3K4me3 demethylation.

Red-light signaling via phytochromes is the most important factor to repress flowering under LD condition. Osugi et al. (2011) showed that phytochromes not only regulate *Ghd7* transcription but also affect Ghd7 protein activity [40]. In addition to the Ghd7 protein, the Hd1 protein is known to act as a suppressor of *Hd3a* under long day-length conditions [12], [41], [42]. Ishikawa et al. (2011) reported that the inhibitory effect of the Hd1 protein on *Hd3a* expression is dependent on *phyB* [43]. Ishikawa et al. (2011) also suggested that the *phyB*-mediated Hd1 suppression of *Hd3a* expression is a component of the molecular mechanism for critical day length in rice [43]. Our results have let us propose a novel flowering repression pathway mediated by red-light signaling. We believe that this pathway of *Se14* will yield crucial information for understanding the daylength dependent repression of flowering in rice.

## Materials and Methods

### Plant Materials

A total of eleven variety/lines, Gimbozu (WT), HS112 (*se14*), HS169 (*ehd1*), HS110 (*hd1*), X61 (*se13*), EG2 (*ghd7*), *ehd1 se14*, *hd1 se14*, *se13 se14*, *ghd7 se14* and SL13, were used. The WT is the *japonica* rice variety Gimbozu. HS112 is an early flowering time mutant line, which was induced by gamma-ray irradiation of WT seeds, and its early flowering has been proven to be caused by a single recessive mutant gene *se14* at the *Se14* locus on chromosome 3 [24]. *ehd1*, *hd1* and *se13* are also flowering time mutant lines, which were created by gamma-ray irradiation of WT seeds, and their differential flowering time is conferred by single recessive mutant alleles, *ehd1, hd1* and *se13,* at the *Ehd1*, *Hd1* and *Se13* locus, respectively [1], [8]. *ghd7* is a tester line for studying flowering time of rice, and its early flowering is controlled by a single recessive allele *ghd7* at the *Ghd7* locus in the Gimbozu genetic background [44]. *ehd1*, *hd1 se14*, *se13 se14*, and *ghd7 se14* are double recessive mutant lines, which were developed from crosses of HS112 with *ehd1*, *hd1*, *se13* and *ghd7*, respectively, and harbor *ehd1*, *hd1*, *se13*, and *ghd7* in addition to *se14*, respectively. SL13 is a chromosome segment substitution line (CSSL) harboring a Kasalath-derived chromosome segment including the candidate region of the *Se14* locus on chromosome 3 on the Nipponbare background (Figure 2A, [26], and the Rice Genome Center: http://www.rgrc.dna.affrc.go.jp). Kasalath is an *indica* rice variety.

### Analysis of Photoperiodic Response

Three single mutant lines, HS112 (*se14*), *hd1*, *ghd7* and WT were used. Ten seeds of each line were sown on field soil filled in a 3.6-L pot and covered with granulated soil. Seedlings were thinned to 5 plants per pot 14 days after sowing, and were grown under four artificial day-length conditions at the green house in Kyoto University, Kyoto, Japan (35°01′N, 135°46′E): 10 h (SD: short day-length), 14.5 h (14.5 LD:14.5 h long day-length), 16 h (16 LD:16 h long day-length) and 24 h (24 LD: continuous light). We also cultivated each plant under natural day-length conditions (ND) at the green house in Kyoto University. In Kyoto, the natural day-length is longer than the critical daylength (approximately 13.5 h) during the cropping season from May to the end of July [25], regarded as a long photoperiod condition, and after that it becomes shorter than 13.5 h regarded as a short photoperiod condition [10]. In addition to natural day length (8∶00–18∶00), supplementary artificial light from incandescent lamps (3.24 Wm^−2^ at soil surface) was used for the 14.5 LD, 16 LD, and 24 LD day-length treatments. The experiment was conducted from 21 May to late October, 2012, with two replications. Flowering time was recorded for each plant when the first panicle emerged from the sheath of the flag leaf.

### Identification of Chromosomal Location and the Candidate Gene of *Se14*

Our previous study showed that the *Se14* locus was located between two markers, RM14315 and MK3_6, on chromosome 3 [24]. To narrow down the candidate region of the *Se14* locus, the F_2_ population, comprising 96 plants from the cross between HS112 and SL13, was grown in a paddy field in Kyoto in 2012. We conducted a fine mapping of the *Se14* locus with SSR (simple sequence repeat) markers and *mPing* sequence characterized amplified region (SCAR) markers [45]. *mPing* SCAR markers are based on polymorphisms of the transposable element *mPing* insertion sites. To further narrow down the region of the mutant gene, we used five SSR markers and one insertion and deletion (INDEL) marker. Flowering time was recorded for each plant when the first panicle emerged from the sheath of the flag leaf. To identify the full-cDNA sequence of *Se14*, 5′-RACE and 3′-RACE were performed with the 5′-full RACE Core Set and the 3′-full RACE Core Set (Takara Bio Inc., Shiga, Japan), respectively, as described in the product instruction manuals.

### Analysis of Interactions of the *Se14* Locus with the *Ehd1*, *Hd1*, *Ghd7*, and *Se13* Loci

Four single mutant lines (*ehd1, hd1*, *ghd7* and *se13*) and four double mutant lines for *se14* (*se14 ehd1, se14 hd1*, *se14 ghd7* and *se14 se13*) were planted under ND conditions in Kyoto in 2012, and examined for flowering time.

### Expression Analysis of Flowering Time in HS112

Plants of HS112 were grown in a cabinet with temperature control under a 14.5 h day length (14.5 h light, 30°C/9.5 h dark, 25°C) at 70% relative humidity. Seedlings were grown on sand with additional liquid fertilizer (Kimura’s B Culture Solution, Nippon Medical & Chemical Instruments Co., Ltd., Osaka, Japan). On the 30 days before flowering, leaves were collected at 4-hour intervals during that day. Total RNAs were extracted with the Trizol reagent (Life Technologies Inc., Gaithersburg, Maryland, USA) according to the manufacturer’s protocols. Total RNA was subjected to DNA digestion by treatment with RNase-free DNase I (Takara Bio Inc.). The Transcriptor first-strand cDNA synthesis kit (Roche Applied Science, Indianapolis, Indiana, USA) was used to reverse-transcribe cDNA from 1 µg of RNA using anchored-oligo(dT)18 primers. Real-time PCR analysis was performed by the Taq-Man PCR method using a LightCycler 1.5 (Roche Applied Science) according to the manufacturer’s instructions. The primer sets of *Hd1*, *Ehd1*, *Hd3a*, *RFT1*, *Ghd7*, *Ehd2* and *UBQ* genes and Universal Probe Library probes of each gene were designed by ProbeFinder version 2.45 (Roche; https://www.roche-applied-science.com/). Primer and Probe sequences are shown in Table S1. Expression analysis using the standard curve method was performed to determine the expression level of each gene. In order to compare expression levels among the genes, the relative expression level of each gene versus the UBQ expression level was calculated. The RNA gene standards for the seven genes were applied to their plasmids prepared by the pGEM-T Easy Vector System (Promega Corp., Madison, Wisconsin, USA) using PCR amplicons from the total RNA of Gimbozu.

### Investigation of H3K4 Methylation State in HS112

Plants of HS112 were grown under a 14.5 h day-length (14.5 h light, 30°C/9.5 h dark, 25°C) at 70% relative humidity conditions. Thirty days after sowing, we collected fully opened leaves from the top of seedlings at two hours after dawn, when the expression of *Ehd1*, *Hd3a* and *RFT1* were changed in HS112. These leaves were chopped into small fragments and infiltrated with 1% formaldehyde for cross-linking and ground in liquid nitrogen after quenching the cross-linking. Chromatin was isolated and sonicated to about 500 bp. Anti-histone H3K4me3 (Abcam Inc., Cambridge, MA, USA) was added to the chromatin solution precleared with magnetic Dynabeads Protein G (Life Technologies Inc.). After subsequent incubation with the Protein G beads, immunocomplexes were precipitated and eluted from the beads. Cross-links were reversed, and residual proteins in the immunocomplexes were removed by incubation with proteinase K followed by phenol/chloroform extraction. DNA was recovered by ethanol precipitation. The amount of immunoprecipitated chromatin of rice flowering genes was determined by real-time PCR on eight different regions of their respective loci using SYBR Green PCR master mix. The primer sets for the ChIP assay, which are listed in Table S2, were made for every 500 bp within the promoter regions and at 5′UTR, 1st exon and 1st intron of *Ehd1*, *Hd3a* and *RFT1*. The amounts of DNA fragments of each region were normalized to the control samples, which were determined by the ChIP assay with H3 universal antibody.

## Supporting Information

Figure S1 **Alignment of deduced amino acid sequences of plant ELF6-like proteins.** This alignment was generated by the ClustalW routine in the MEGA 5 software (Tamura et al. 2011). The alignment is presented by GENEDOC (www.psc.edu/biomed/genedoc). Residues on black and gray backgrounds indicate 100% and 60% amino acid similarity, respectively. Conserved (black), similar (gray) and non-conserved amino acid residues (white) were highlighted with the GENEDOC software. A dotted line, black line and boxes indicate JmjN, JmjC and ZnF domains, respectively. A triangle indicates the mutation site in HS112. Species abbreviations and sequence IDs: *Oryza sativa Se14*: Os03g0151300 and Os03g0151400 (LOC_Os03g05680.1 and LOC_Os03g05690); *Arabidopsis thaliana ELF6*: AT5G04240.1; *O. sativa ELF6-like gene*: Os12g0279100 (LOC_Os12g18150.1); *Glycine Max GmELF6*: XP_003555439; *Lotus japonicus LjELF6*: Chr5.LjT17D03.90.r2.d; *Zea mays ZmELF6*: ZEMMB73_57839. Accession and locus numbers correspond to GenBank IDs.
(TIF)

Figure S2 **Diurnal expression of** ***Se14*** **in WT and the expression of** ***Se14*** **in the** ***Se13*** **deficient mutants.** Diurnal expression analysis was performed by the standard curve method. For comparing expression levels among the genes, the relative expression level of *Se14* against the *UBQ* expression level was calculated. WT and the *Se13* deficient mutant were grown under 14.5 h day-length conditions. Leaves of three plants were sampled at 4 h intervals (three replications) 30 days after sowing. The black bars indicate the dark period, and the white bars indicate the light period.
(TIF)

Table S1 **Primer sets for expression analysis.**
(TIF)

Table S2 **Primer sets for ChIP assay.**
(TIF)
