# Supplementary figures and images for: Se14, Encoding a JmjC Domain-Containing Protein, Plays Key Roles in Long-Day Suppression of Rice Flowering through the Demethylation of H3K4me3 of RFT1
Source: PLoS One. 2014 Apr 23;9(4):e96064. doi: 10.1371/journal.pone.0096064 (PMC3997562; doi:10.1371/journal.pone.0096064)

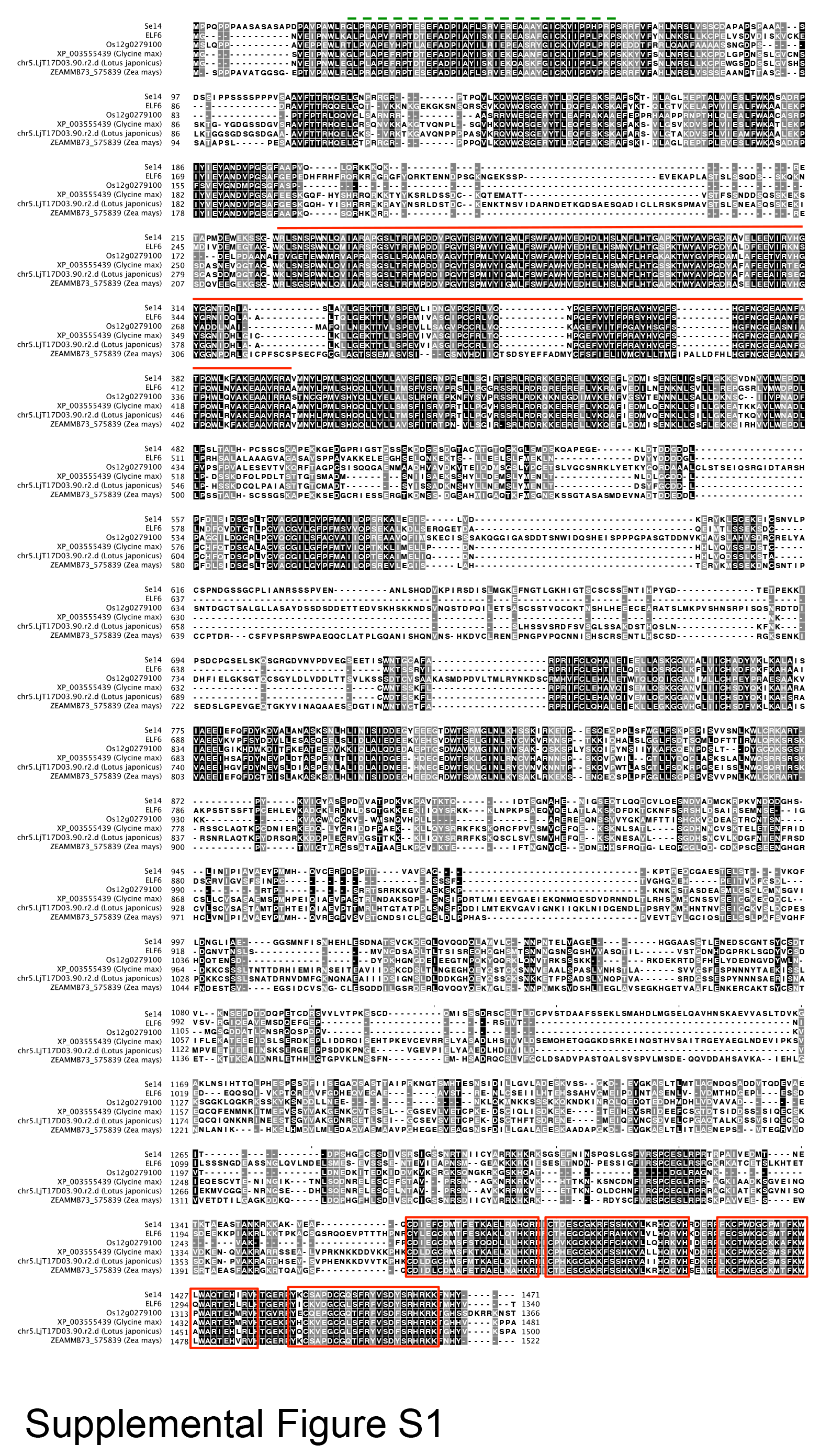

Supplement: Figure S1 — Alignment of deduced amino acid sequences of plant ELF6-like proteins. This alignment was generated by the ClustalW routine in the MEGA 5 software (Tamura et al. 2011). The alignment is presented by GENEDOC (www.psc.edu/biomed/genedoc). Residues on black and gray backgrounds indicate 100% and 60% amino acid similarity, respectively. Conserved (black), similar (gray) and non-conserved amino acid residues (white) were highlighted with the GENEDOC software. A dotted line, black line and boxes indicate JmjN, JmjC and ZnF domains, respectively. A triangle indicates the mutation site in HS112. Species abbreviations and sequence IDs: Oryza sativa Se14: Os03g0151300 and Os03g0151400 (LOC_Os03g05680.1 and LOC_Os03g05690); Arabidopsis thaliana ELF6: AT5G04240.1; O. sativa ELF6-like gene: Os12g0279100 (LOC_Os12g18150.1); Glycine Max GmELF6: XP_003555439; Lotus japonicus LjELF6: Chr5.LjT17D03.90.r2.d; Zea mays ZmELF6: ZEMMB73_57839. Accession and locus numbers correspond to GenBank IDs. (TIF) [file pone.0096064.s001.tif]

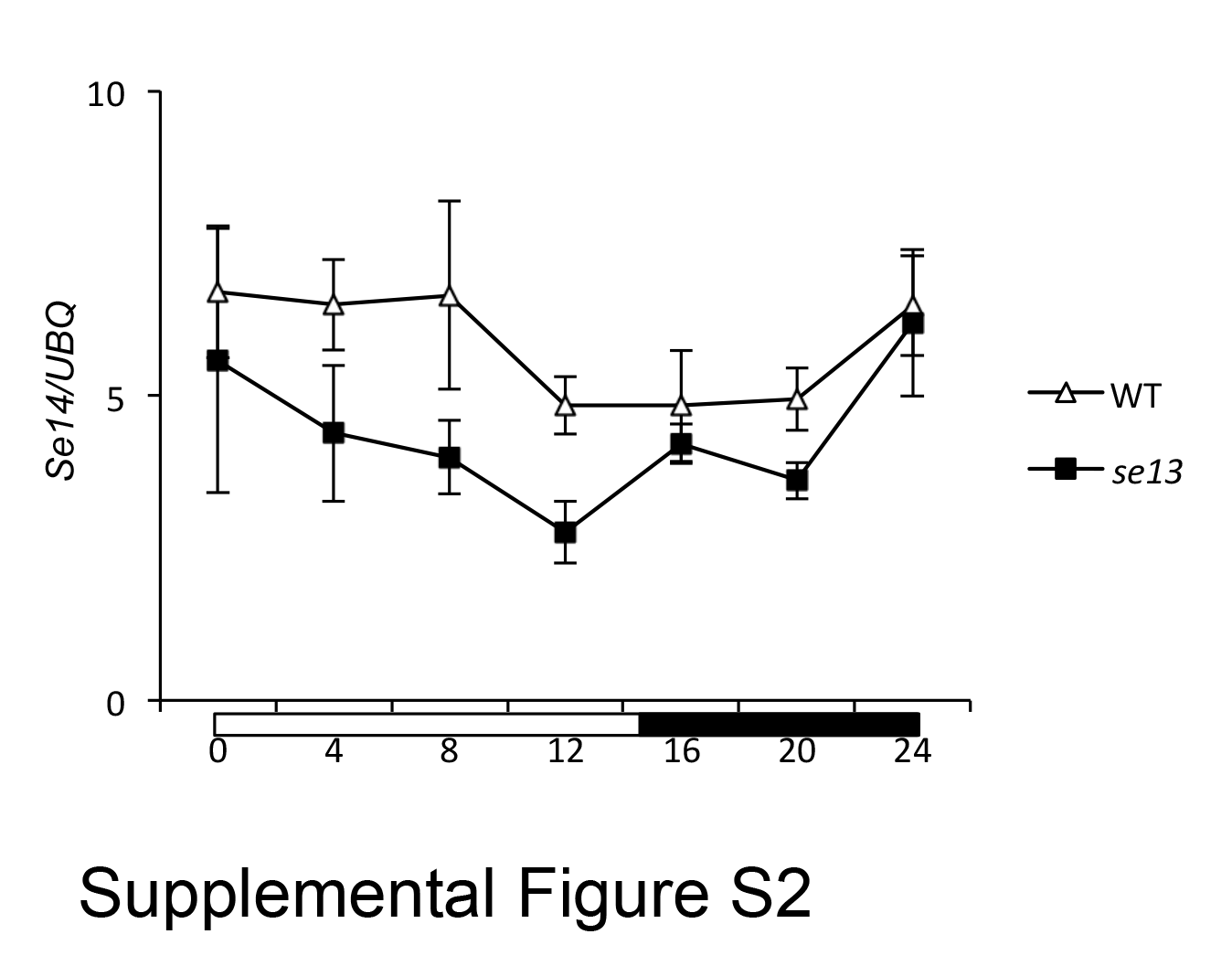

Supplement: Figure S2 — Diurnal expression of Se14 in WT and the expression of Se14 in the Se13 deficient mutants. Diurnal expression analysis was performed by the standard curve method. For comparing expression levels among the genes, the relative expression level of Se14 against the UBQ expression level was calculated. WT and the Se13 deficient mutant were grown under 14.5 h day-length conditions. Leaves of three plants were sampled at 4 h intervals (three replications) 30 days after sowing. The black bars indicate the dark period, and the white bars indicate the light period. (TIF) [file pone.0096064.s002.tif]

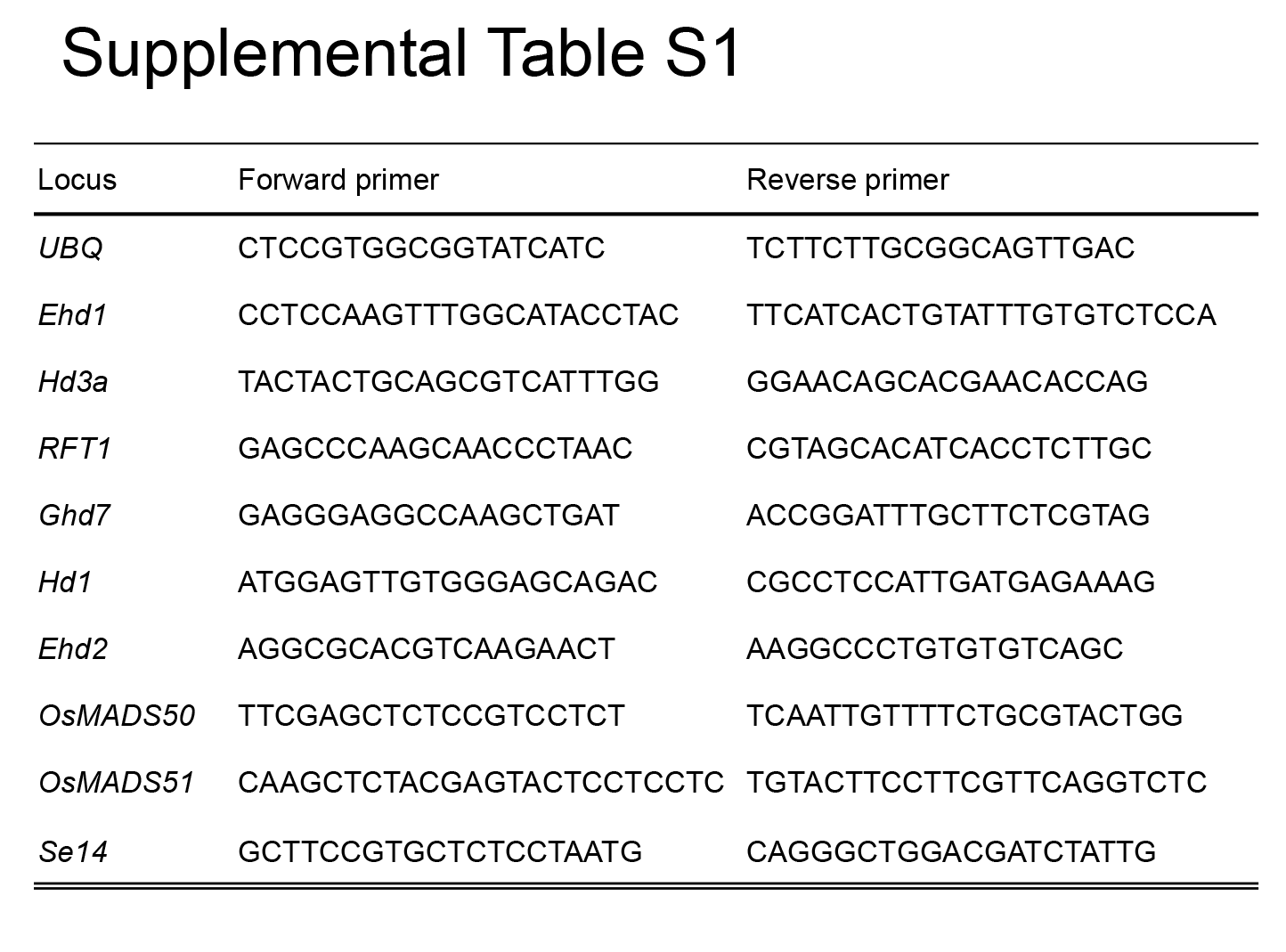

Supplement: Table S1 — Primer sets for expression analysis. (TIF) [file pone.0096064.s003.tif]

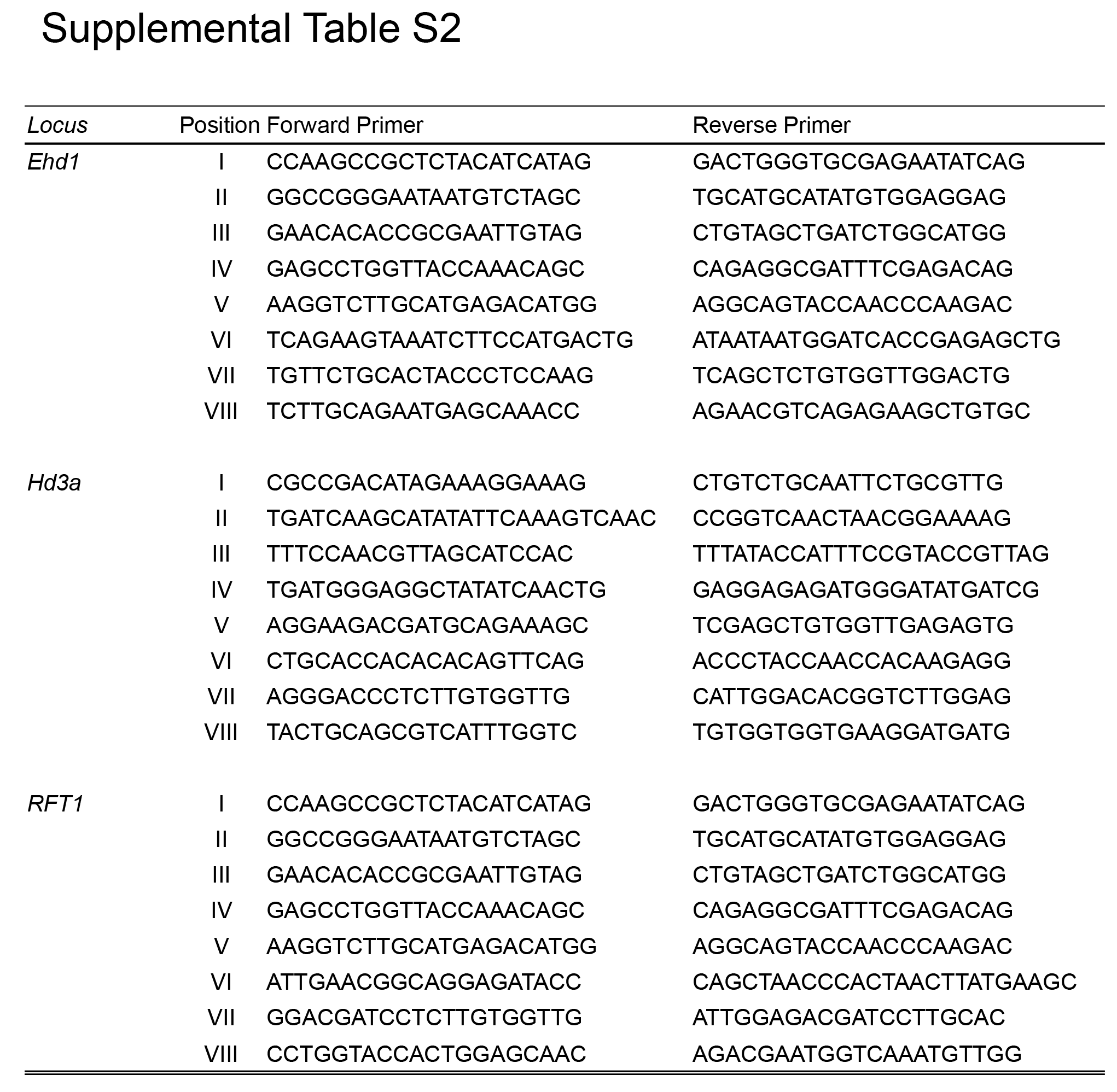

Supplement: Table S2 — Primer sets for ChIP assay. (TIF) [file pone.0096064.s004.tif]
